# Early cochlear implantation in prelingual profound hearing loss in Italy, analyzed by means of a social media survey

**DOI:** 10.3389/fped.2023.1031341

**Published:** 2023-02-03

**Authors:** Eva Orzan, Giulia Pizzamiglio, Jad Magadle, Luciano Bubbico, Jodi M. Cutler, Patrizia Consolino, Sandro Burdo, Giulia Zamagni, Elena Magni, Claudio Mariottini, Valeria Gambacorta, Giampietro Ricci, Davide Brotto

**Affiliations:** ^1^ Institute for Maternal and Child Health IRCCS “Burlo Garofolo”, Trieste, Italy; ^2^ Department of Radiology, University of Trieste, Trieste, Italy; ^3^ Neurology Department, Hadassah University Hospital (Ein Kerem), The Hebrew University, Jerusalem, Israel; ^4^ Department of Sensorineural Disabilities, INAPP/Italian Institute of Social Medicine, Rome, Italy; ^5^ NPO Associazione ASI Affrontiamo la Sordità Insieme, Carpi, Italy; ^6^ ENT Department, Martini Hospital, Turin, Italy; ^7^ NPO Associazione Italiana Liberi di Sentire, Varese, Italy; ^8^ NPO Famiglie Associate per la Difesa dei Diritti Degli Audiolesi Dell'Umbria (FIADDA Umbria), Castiglione del Lago (Perugia), Italy; ^9^ Department of Surgery and Biomedical Sciences, Section of Otorhinolaryngology, University of Perugia, Perugia, Italy; ^10^ Otorhinolaryngology Section, Neurosciences Department, Università di Padova, Padova, Italy

**Keywords:** newborn hearing screening, profound hearing loss, social media, cochlear implant, hearing loss

## Abstract

**Objective:**

To assess newborn hearing screening (NHS) impact on timing of cochlear implant (CI) surgery of patients with prelingual bilateral profound hearing impairment (BPHI), in order to evaluate whether the NHS ultimately serves the needs of the target population in Italy.

**Methods:**

An online questionnaire was created to survey subjects affected by prelingual BPHL born between 1990 and 2018. Questions focused on age at BPHI diagnosis, first and second CI surgery (if performed), and the region in which the surgery was performed. The survey was distributed to potential participants *via* social media communities used by hearing impaired people or their family members for sharing advice and offering support. Responses were analyzed using descriptive statistics.

**Results:**

Among the 318 respondents who completed the questionnaire, 276 (87%) reported having chosen CI surgery, 2/3 of them bilaterally. In the vast majority (97%) of cases the CI is used on a daily basis. Most of the people residing in the center (65%) and southern Italy (71%) had to move from their region of residence to perform the surgery. Late CI surgery was associated with failure to perform NHS (*p* = 0.007), birth before 2011 (*p* = 0.009), definitive diagnosis of BPHI after 6 months of life (*p* = 0.002), and progressive hearing impairment (*p* < 0.001).

**Conclusion:**

The worldwide scientific approval of the NHS as the current best opportunity for early diagnosis and CI treatment for prelingual BPHI is confirmed by what patients and families reported *via* the online questionnaire used for this study. In recent years, early bilateral cochlear implantation has become increasingly available in Italy, but late diagnosis, progressive hearing loss, failure to perform the NHS and lack of follow-up are still open questions. A large proportion of families had to move from the region of residence to have their child undergo CI surgery, revealing inequalities in terms of geographical disparities. Social media has proved to be a valuable, fast and inexpensive tool for gathering information on the effectiveness of health prevention programs, involving a large sample of individuals in a short amount of time.

## Introduction

Newborn hearing screening (NHS) has been identified as a critical step in achieving early hearing impairment diagnosis and treatment (1), and cochlear implants (CI) are now universally considered to be the standard of care for the medical treatment of bilateral profound sensory-neural hearing impairment (BPHI) in children. Cochlear implantation restores the sense of hearing and access to the sounds of speech offering the opportunity to develop receptive and expressive spoken language skills that were previously not achievable for the vast majority of children with BPHI. Early surgery is feasible and safe even in the first months of life (2). In addition, simultaneous bilateral cochlear implantation has become widely available in recent years to ensure the shortest duration of hearing deprivation and the best audiological outcomes (3). In all cases, a complete audiological evaluation and a complete radiological work-up are mandatory for a safe surgical procedure (4).

According to data collected by the Italian Ministry of Health, in the last 10 years about a thousand CIs have been performed every year in Italy, with a constant growth trend (5). While multiple centers in the Italian Health System currently offer cochlear implantation opportunities, the vast majority of these procedures are performed in hospitals located in the northern part of the country.

Unfortunately, only scattered data about some regions of Italy (i.e., Tuscany (6) and Sicily (7)) are available about the system of NHS and a complete representation of the national system is missing in current literature (8).

Consequently, an online survey was conducted to analyze the effectiveness of the NHS in guaranteeing early intervention for patients with BPHI. This low-cost survey methodology allows for the collection of a large cohort of patients in a relatively short period of time (9–11). Specifically, the purpose of this study was to collect information on the performance, timing and geographical location of the surgery, the uni- or bilateral intervention and CI use, investigating its possible relationship with NHS performance and outcome. Much of the available clinical literature has focused on the efficacy of NHS and CI (1, 12). Conversely, less research has been devoted to investigating how these aspects are captured from the perspective of patients, which is the aim of the present study.

## Material and methods

An online survey titled “Screening Uditivo Neonatale Universale: la promessa viene mantenuta? Un’indagine attraverso i Social Media ‘‘(English title: “Universal newborn hearing screening: Are We Keeping the promise? A social media survey”) has been designed by the Otorhinolaryngology and Audiology Unit of the Institute for Maternal and Child Health IRCCS “Burlo Garofolo'‘ of Trieste, Italy. A specific questionnaire was developed by a group of health operators including medical doctors specialized in audiological medicine, audiologists, speech therapists and by representatives of patients” associations online forums with specific expertise and interest in NHS, hearing loss and cochlear implantation. The questionnaire aimed at investigating how the NHS is impacting on early diagnosis and treatment of BPHI, from the patient's perspective.

The survey included 30 multiple choice items, divided into three parts and a final open question. The present paper mainly discusses the results of the second part of the questionnaire that took into account data about cochlear implant recipients, such as where surgery was performed, the timing of first and second CI, if performed. The last part regarded other basic information on rehabilitation, education and other health service facilities. Finally, an open question gave the possibility to patients and families to describe their personal journey regarding BPHI identification, investigation and treatment.

The questionnaire was available only in Italian. The estimated time required to complete the questionnaire was estimated to be 10–15 min.

Prior to online publication, the questionnaire was administered to 20 patients and/or their parents (in case of patients too young to be actively involved). The aim was to detect any critical issues or difficulties in understanding and to identify any obstacles to completing the questionnaire. The questions were subsequently revised according to this preliminary test.

The online version of the questionnaire was created by means of the “Survey Administration App” on the web platform Google Forms, and shared *via* several web channels (the Facebook groups “Affrontiamo La Sordità Insieme: forum impianto cocleare” and “Portatori Impianti Cocleari Italiani”, and shared within mailing lists of the largest Italian dedicated associative groups, “FIADDA Onlus”, “FIADDA Umbria Onlus”. “Associazione Liberi Di Sentire Onlus” and “Ciao Ci Sentiamo - Onlus”). The platform allows 1) to restrict the time of online publication, 2) to limit access for each account, 3) to maintain anonymity 4) to provide complete information and 5) to receive consent for the use of data for medical research.

The questionnaire has been posted online for 3 months (between March and June 2019) and periodically shared as a link *via* the above-mentioned web channels. A copy of the questionnaire is available as online material (see Supplementary Material).

The call aimed to include subjects affected by a congenital or prelingual bilateral BPHI (mean hearing threshold > 80 dB HL in the best hearing ear) born from 1990 to 2018, assuming that these patients should have completed their diagnostic and therapeutic process. It should be considered that some of the patients born in 2018 were most probably too young to have undergone CI surgery at the time of the survey. Since the first regional experiences of the NHS in Italy date back to 1997 (13), the wide time span was chosen to compare the pre and post NHS era.

The data were completely collected before COVID-19 pandemic.

This research was funded by I.R.C.C.S. “Burlo Garofolo.”, grant number RC 42/22.

All parents or caregivers of subjects eligible for the study were informed of the objective by the study evaluators and, they gave their consent by answering the questionnaire.

The study was conducted in accordance with the 1964 WMA Helsinki declaration and its later amendments, under the framework of the research project RC 42/22 approved by the institutional ethical review board, nominated by the Italian Ministry of Health (Ufficio per la Ricerca Clinica IRCCS Burlo Garofolo).

### Statistical analysis

The percentages of newborns screened on a nationwide basis in the reporting years were analyzed and compared. The difference between categorical variables was evaluated using the Fisher exact test. *P* values <0.05 were considered significant.

**Figure 1:**
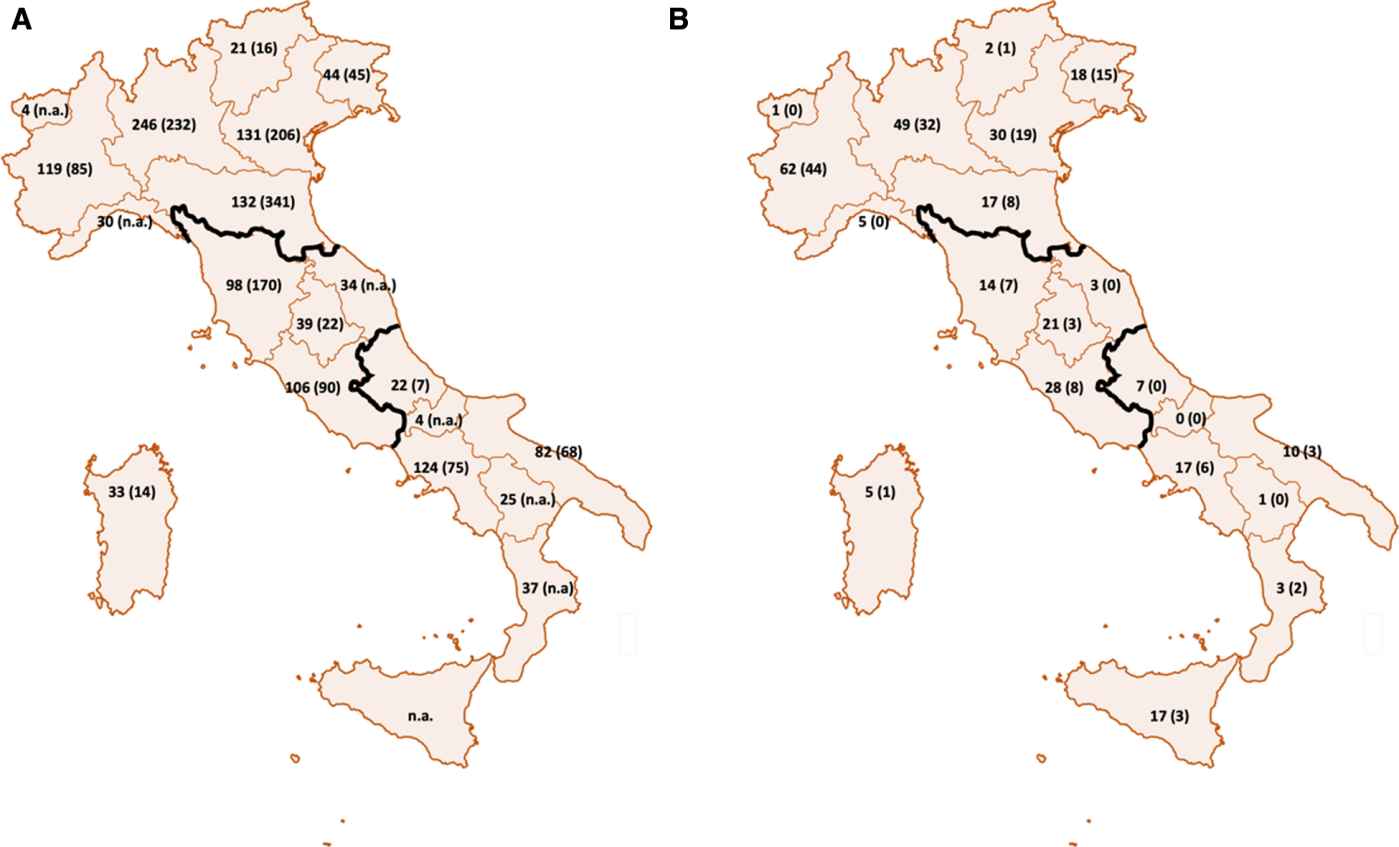
Geographical localization of CI recipients in Italy and in our cohort. In figure (**A**) are presented the number of patients needing a cochlear implant for each region and the number of procedures (between parenthesis) performed in 2018 in Italy. The data are obtained by the surveillance program of the Italian Ministry of Health. In figure (**B**) are presented the number of patients collected in our cohort that were born from each region and the number of patients who performed the CI in the same region (between parenthesis).

Additional analysis was performed by clustering the geographical data in three areas (see Figure 1): Northern Italy (Friuli Venezia Giulia, Veneto, Trentino Alto Adige, Lombardy, Piedmont, Aosta Valley, Liguria, Emilia Romagna), Central Italy (Tuscany, Marche, Umbria, Lazio) and Southern Italy (Abruzzo, Basilicata, Campania, Molise, Puglia, Calabria, Sicily, Sardinia).

Logistic regression was used for the evaluation of the potential risk factors for the surgical delay of implantation, such as NHS outcome, age of diagnosis, progressiveness, BPHI features, multiple disabilities, region of origin, the change of region for surgery and the period of birth.

All analyses were conducted by using StataCorp 2019 Stata Statistical Software: Release 16, College Station, TX: StataCorp LLC.

## Results

A total of 332 questionnaires were received. The check for completeness and coherence of the answers led to include 318 questionnaires related to subjects affected by congenital or prelingual PHL. Among the 318 respondents, 276 (87%) patients reported having undergone CI surgery (42 did not undergo CI surgery). In 119/276 (43%) of cases, the surgery for the first CI was performed outside the region of residence: this was the case for the majority of respondents living in the Southern (37/52, 71%) or Central (33/51, 65%) part of Italy. Only 45/165 (27%) of subjects residing in the Northern regions had to move for the surgery (1 patient did not answer the question). Four out of eight patients born abroad received their CI in a foreign country before moving to Italy.

At the time of the questionnaire, 95/276 (34%) subjects reported using a unilateral, while 181/276 (66%) a bilateral CI. Almost all subjects (269/276, 97%) use the CI on a daily basis.

**Figure 2:**
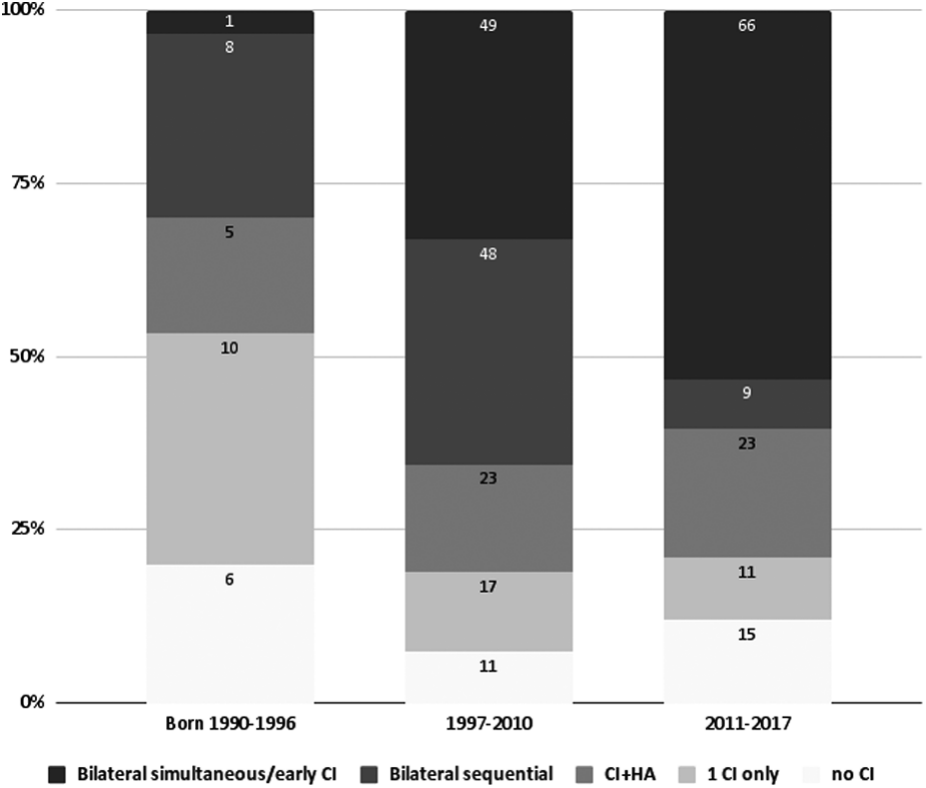
Ci choices in our cohort from 1990 to 2017. Respondents were clustered into three periods and are indicated on the x-axis (1990-1996 pre NHS era, 1997–2010 era of first scattered applications of NHS, 2011-2017 NHS era). The CI rehabilitative choices have been defined as follows: no CI (the person does not use any CI), unilateral CI (the CI has been applied only on one side, no hearing device is used on the other side); CI + HA (bimodal stimulation, with CI on one side and a hearing aid on the contralateral ear); bilateral sequential (if the CIs have been applied with a time interval greater than 1 year between the first and second surgery); bilateral simultaneous/early (if bilateral CIs have been applied simultaneously or in any case within 12 months of time span). Patients born in 2018 were most probably too young to have undergone CI surgery at the time of the survey and were therefore excluded from the chart.

For subsequent analysis, subjects undergoing simultaneous bilateral CI surgery were clustered with patients who performed the contralateral surgery within 12 months of the first CI. In the case of bilateral CI, simultaneous/early surgery was performed in 117 cases (65% of bilateral CIs) and late sequential surgery in 64/185 cases (35%). Among patients with unilateral CI, 43/95 (45%) did not report the use of any contralateral hearing aid. Figure 2 shows the treatment choices: to better appreciate the progress in the choice of treatment modality, the respondents were divided into groups based on the year of birth excluding patients born since 2018 probably too young to have undergone CI surgery at the time of the survey.

Considering the entire cohort of patients with CI, surgery occurred within 12 months of life in 60/276 (21.7%) CI recipients, of which 44/60, (73%) had a simultaneous or early bilateral sequential surgery. In 93/276 subjects (33.7%), the first CI intervention was performed between 13 and 24 months of age (of which 43/93, 46.2% simultaneous/early bilateral), in 31/276 (11, 2%) between 25 and 36 months (of which 10/31, 32.3% simultaneous/early bilateral), and finally in 92/276 (33.3%) cases it was performed after the 3rd year of life (of which 20/92, 22% simultaneous/early bilateral).

**Figure 3:**
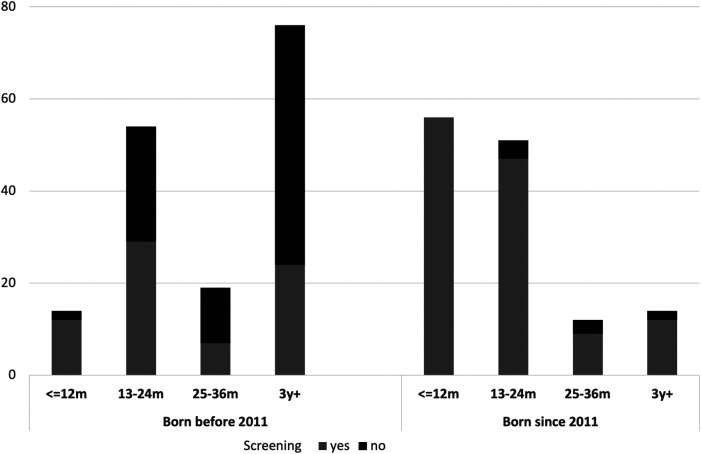
Barplot of the cohort of the study. Age at first (or bilateral simultaneous) CI surgery in relation to the period of birth (before and after 2011) and the performance of NHS at birth. The execution of NHS influences significantly the age at first CI surgery (*p* < 0.001) and a decrease in the age of the first CI surgery can be observed for those born since 2011 (*p* < 0.001).

Figure 3 shows the difference in age at the first CI surgery based on whether the NHS was performed or not (*p* < 0.001) and the different age of first CI surgery if the two birth periods considered are compared. (pre and post 2011, *p* < 0.001).

Comparing the results of early CI with early, late and no CI, clustered for the geographical origin, a statistical difference was observed between patients from the Northern and those from Central and Southern Italy (*p* < 0.05).

On the other hand, while comparing early CI with the group including early and late CI, 103/126 (82%) were from Central and Southern Italy, while 165/184 (90%) from Northern Italy. In this case, only a trend towards significance can be observe with a Fisher exact test (*p* = 0.062).

In order to detect the presence of other issues that could affect early on the earliness of the CI intervention, multiple factors were examined for the 153 cases for whom the first CI surgery was performed before 24 months, and the 123 cases of CI surgery performed after 24 months of age.

Predictive factors for late cochlear implantation in the study population are presented in Table 1A. By multivariate analysis, progressive hearing loss (OR 5.76; 95% CI 2.78–11.94; *p* < 0.001) and diagnosis after 6 months (OR 3.12; 95% CI 1.53–6.40; *p* < 0.002) are the main independent clinical predictors of late cochlear implantation. The introduction of NHS has resulted in a clear benefit in the early treatment of congenital PHL, in fact, on multivariate analysis, being born from 2011 onwards is a favorable factor for early implantation (OR 0.40; 95% CI 0.20–0.80; *p* = 0.009), just as not having NHS delays implantation (OR 2.88; 95% CI 1.33–6.24; *p* = 0.007).

**Table 1:** Univariate and multivariate logistic regression for the evaluation of potential risk factors for the surgical delay of cochlear implantation.

(**A**)								
			Univariate			Multivariate		
	Early CI (*N* = 153)	Late CI (*N* = 123)	OR	95% CI	*p*-value	OR	95% CI	*p*-value
**UNHS outcome**								
Refer	110 (72)	35 (28)	–	–	–	–	–	–
Pass	14 (9)	17 (14)	3.82	1.71–8.52	***0***.***001***	1.93	0.71–5.29	0.199
No/Unknown	29 (19)	71 (58)	7.69	4.33–13.68	***<0***.***001***	2.88	1.33–6.24	***0***.***007***
**PHL diagnosis**								
Before 6 months	99 (65)	24 (19)	–	–	–	–	–	–
After 6 months	54 (35)	97 (79)	7.41	4.25–12.93	***<0***.***001***	3.12	1.53–6.40	***0***.***002***
Unknown	0	2 (2)	–	–	–	–	–	–
**Progressive HI**								
No	122 (80)	55 (45)	–	–	–	–	–	–
Yes	23 (15)	55 (45)	5.30	2.97–9.49	***<0***.***001***	5.76	2.78–11.94	***<0***.***001***
Unknown	8 (5)	13 (10)	3.60	1.41–9.20	***0***.***007***	2.00	0.68–5.90	0.209
**HI features**								
Isolated	130 (85)	101 (82)	–	–	–	–	–	–
Non isolated	23 (15)	22 (18)	1.23	0.65–2.33	0.524	1.45	0.63–3.37	0.383
**Region of origin**								
North	101 (66)	64 (52)	–	–	–	–	–	–
Center	25 (16)	26 (21)	1.64	0.87–3.09	0.124	1.88	0.81–4.39	0.142
South	22 (15)	30 (25)	2.15	1.14–4.05	***0***.***018***	1.90	0.82–4.41	0.132
Abroad	5 (3)	3 (2)	0.95	0.22–4.10	0.942	1.29	0.22–7.53	0.774
**Changed region for surgery**								
No	97 (63)	58 (47)	–	–	–	–	–	–
Yes	55 (36)	64 (52)	1.95	1.20–3.16	***0***.***007***	1.23	0.64–2.35	0.537
Unknown	1 (1)	1 (1)	–	–	–	–	–	–
**Year of birth**								
1990–2010	66 (43)	95 (77)	–	–	–	–	–	–
2011–2018	87 (57)	28 (23)	0.22	0.13–0.38	***<0***.***001***	0.40	0.20–0.80	***0***.***009***

**Table:** 

(**B**)				
Age of identification PHL	With other pathologies (%)		Without other pathologies (%)	
	Screened	Not screened	Screened	Not screened
0–6 months	25 (45)	2 (4)	103 (40)	13 (5)
> 6 months	15 (27)	13 (24)	63 (24)	81 (31)

Early CI: unilateral or bilateral simultaneous surgery performed within 24 months of age; Late CI = unilateral or bilateral simultaneous surgery performed after 24 months of age.

*χ2 = 109.15, DF = 11, PseudoR2 = 0.2911, *p*-value < 0.001.

In the univariate analysis, being from southern Italy (OR 2.15; 95% CI 1.14–4.05; *p* = 0.018) and having to change region for surgery (OR 1.95; 95% CI 1.20–3.16; *p* = 0.007) are risk factors for late cochlear implantation, although they do not emerge as independent factors in the multivariate analysis. Finally, the age of implantation was not influenced by the presence of associated pathologies (*p* = 0.524). (Table 1B).

## Discussion

The present study, based on an online survey, underlines - through the reports of the patients themselves - the significant impact of the NHS performance on the early treatment of congenital BPHI in Italy. Also, the online surveys have proved to be an extremely useful tool for actively involving patients in evaluating the effectiveness of the NHS.

As previously reported for other countries (14), the data from the present study confirm the geographic disparity not only in diagnostic terms, but also in terms of access to CI treatment for congenital BPHI. In fact, more than 60% of patients born in central-southern Italy moved to a different region for CI surgery, while less than 30% of patients in the North did. Indeed, more than 60% of patients born in Central or Southern Italy moved to a different region for CI surgery, while less than 30% of Northern Italy patients did. The data reported by the patients are in line with those collected by the Italian Ministry of Health (5). Indeed, by comparing the number of CI interventions and the number of resident patients who have undergone CI surgery for each region, the statistics reveal a negative balance ((-163)) for Southern Italy and a positive balance (+198) for the North (5).

In particular, the chance of an early CI surgery is not significantly different between Northern and Central and Southern (combined) Italy, but a trend towards significance is present favoring patients from the Northern part of the country. This difference may be due to the fact that the Northern part of the country presents a higher number of tertiary centers, a higher mean personal income and a higher population density compared to the rest of the country.

Considering the limited chance to perform NHS and the necessity to move for CI surgery, the overall data highlight a general frailty of the health care system of Central and Southern regions of Italy. Even if the opportunities for the patients seemed to be improved over time, an important disparity is present and perceived by CI recipients still today. It should be remembered that the United Nations Committee on the Rights of the Child and Adolescent recommend constant monitoring and service. This is necessary to minimize and prevent disabilities and to overcome territorial differences in essential levels of care for all children regardless of where they are born (13, 15). This goal should also be pursued within each country.

Analyzing the whole cohort of respondents, around 90% of subjects underwent cochlear implantation (and almost the whole cohort uses the implant on a daily basis): it could be inferred that CI is a widely accessible procedure in Italy, although there are still a number of children with profound hearing loss who may not be adequately treated or who forgo IC surgery.

One third of patients with BPHI reported having received unilateral CI; this proportion represents half of the cohort of those born between 1990 and 1996. Simultaneous and sequential bilateral cochlear implantation has been reported more frequently (60% of patients), especially in recent years. Most likely, this can be explained by recalling that cochlear implantation has been an FDA-approved procedure in childhood only since the 1990s, and became available worldwide in the following decades, initially as a unilateral surgery. The surgical opportunity and auditory advantages of the contralateral CI only emerged later (16, 17) As it can be easily observed by the present results (see Figure 2), the number of bilateral simultaneous/early CI patients increases over time, thus confirming the above-mentioned considerations.

Around a half of patients had their first CI surgery within the first two years of life. This is in line with the literature that highlights that CI surgery should be performed within the first 24 months of age in order to obtain the best auditory outcome (18, 19), even if other studies suggest that the best results can be obtained within the 9–12 months age (20, 21). The present results also reveal that a significant association is present between the age of first CI both with the performance of the NHS and the period of birth. Moreover, the definitive diagnosis of BPHI after the 6th month of life is significantly related to a late CI surgery. These data are in line with the most recent literature about the NHS collected with different approaches (22, 23).

In clinical practice, comorbidities appear to be the main reason for delaying the surgical procedure (24) However, in our series this is not confirmed: from the analysis of our data, the timing of CI surgery was not influenced by the presence of a multiple disability. This may be due to the method used for the data collection, the limited cohort considered or to the selection bias of the patients. Concomitant health issues (such as cardiac malformations) are life-threatening conditions requiring prompt intervention, consequently the treatment of hearing loss may be reasonably delayed in such cases, even for anesthetic considerations (25).

On the other hand, CI is a feasible procedure to manage even in the presence of concomitant pathologies, so it is possible that modern clinical and surgical management may have reduced the difference between patients with isolated and syndromic hearing loss.

Patients with progressive BPHI less frequently undergo CI surgery and the procedure is performed later in time. These data highlight that the management of these patients remains difficult. Often a comprehensive audiological evaluation of progressive impairment requires multiple steps in tertiary care hospitals in order to achieve a definite diagnosis and to promote a prompt habilitation of the child. It is common that patients (and their families) have to move to specialized pediatric audiology care centers for an in-depth evaluation of the auditory disorder, for the genetic counseling and testing, for radiological studies and for cochlear implantation.

The concomitant occurrence of a progressive hearing loss, the presence of multiple disabilities, the socioeconomic boundaries and the geographical distances can be influential factors in delaying the early habilitative intervention in BPHI subjects. An appropriate communication between clinicians and families may help in reducing the potential difficulties that patients living in underdeveloped areas might face. Clinicians involved in the NHS should also highlight the importance of the timing for the diagnosis and treatment that can prevent future problems in terms of language, neurocognitive, social and emotional development. The abovementioned impairments might result in long term consequences such as insufficient academic outcomes and reduced professional opportunities.

It should be noted that the data collected should not be intended as being representative of the entire Italian population with BPHI. The questionnaire was initially disclosed among associations and forums that support the prosthetic treatment of deafness, even if during the stay on the network it was presumably shared also among subjects with BPHI who chose not to opt for the CI.

Some limitations of this study should be mentioned.

First, the questionnaire was specifically developed for the present study and it was not previously validated.

Second, the technology used itself selects patients who can access IT infrastructures.

Third, the respondents are part of social media representative groups. Consequently, the present cohort is composed of motivated subjects intrinsically more involved in healthcare issues and therefore these patients might not be necessarily representative of the entire population under study. Fourth, the clinical data are obtained *via* patients' answers, so their quality depends on the patient's ability to understand and remember his/her medical history.

Finally, data collection dates back to 2019. Although this might be seen as a limit, this is also a merit, as they represent the overall course of the treatment of the patients, without the impairment of specific influential factors. In addition, these data will be useful for a comparison with data of patients born during pandemic, so we will be able to assess the impact of this event in the natural history of hearing loss management.

For sure, the results would have had a different scientific weight if derived from questionnaires administered directly in the implant centers.

Despite all of this, the above-mentioned data seem to be coherent with the previously reported literature on UNHS. Consequently, the present study based on this new methodology takes on an informative significance regarding the relationship between neonatal screening and early diagnosis of PHL.

Based on a wider view, healthcare systems are internationally encouraged to increase the audiology evaluation service's capacities, the level of professional knowledge among audiology professionals, the access to the service for families, the information flows, the data management and the hearing surveillance in childhood related to NHS (26). Italy, as well as other countries (27), is currently facing these problems aiming to reduce the possible inequities with national laws to limit the above-mentioned differences in terms of regional application of the NHS (28), to improve the rate of identification of patients affected by hearing loss (29) and to achieve an early treatment of these patients.

## Conclusions

The worldwide scientific endorsement of NHS adherence, as being the current best opportunity for early diagnosis and cochlear implant surgery diagnosis for patients with congenital BPHI, seems to be confirmed by what was reported by patients *via* the online questionnaire used for the present study. Although a large proportion of families have to change regions to have their child undergo surgery in cases of preverbal BPHI, cochlear implantation is widely accessible in Italy. Early bilateral implantation is increasingly performed as it appears to provide the best opportunities for hearing development (30).

In order to reduce the time span between BPHI diagnosis and surgery, the close relationship between birth centers where NHS is performed and pediatric audiology care centers capable of early cochlear implantation should be further strengthened. In short, our data suggests that we are close enough to deliver on the NHS “promise” of early intervention, even if the organizational commitment is not yet concluded.

## Data Availability

The raw data supporting the conclusions of this article will be made available by the authors, without undue reservation.
